# Community engagement strengthens pig disease knowledge and passive surveillance in Timor-Leste

**DOI:** 10.3389/fvets.2022.1024094

**Published:** 2023-01-12

**Authors:** Joanne Millar, Olavio Morais, Henriqueta Da Silva, Paul Hick, Ayrial Foster, Joanita Bendita da Costa Jong, Abrao Pereira, Shawn Ting, Felisiano da Conceição, Jenny-Ann L. M. L. Toribio

**Affiliations:** ^1^Gulbali Institute, Charles Sturt University, Albury, NSW, Australia; ^2^National Directorate of Veterinary, Ministry of Agriculture and Fisheries, Dili, Timor-Leste; ^3^Qualitative Research Facilitator and Consultant, Dili, Timor-Leste; ^4^Elizabeth Macarthur Agricultural Institute, New South Wales Department of Primary Industries, Menangle, NSW, Australia; ^5^Berrimah Veterinary Laboratory, Department of Primary Industry and Resources, Northern Territory Government, Darwin, NT, Australia; ^6^Global and Tropical Health Division, Menzies School of Health Research, Charles Darwin University, Darwin, NT, Australia; ^7^Sydney School of Veterinary Science, Faculty of Science, The University of Sydney, Camden, NSW, Australia

**Keywords:** community engagement, biosecurity, pig health, disease surveillance, Timor Leste

## Abstract

Smallholder pig production in Timor-Leste is culturally and economically important for most households. However, regular and ongoing disease surveillance and pig husbandry training for farmers are limited. This article describes collaborative social and diagnostic research followed by a pilot community engagement program to improve farmer and technician knowledge, skills, and working relationships. There were three phases: (1) A qualitative study in 2020 to explore the experiences and knowledge of 133 pig farmers, 6 village leaders, and 16 district veterinary technicians on pig diseases and reporting, treatment methods, and access to information or assistance. (2) A pilot community engagement program in 3 villages in 2021 with the diagnostic investigation with samples analyzed from 27 dead pigs, and (3) Evaluation of community engagement and training outcomes. Results of the qualitative study revealed limited reporting of sick or dead pigs by farmers to veterinary technicians due to a lack of trust in the veterinary diagnostic system. Most technicians lacked experience with sampling or post-mortems so diagnostic training was undertaken for the pilot disease investigation. Evaluation results showed improved knowledge, motivation, and confidence of government staff and farmers. The credibility of veterinary technicians improved and gave them more confidence to work with communities. Farmers felt supported because all aspects of pig husbandry were addressed, and they were more willing to report dead or sick pigs. The project indicates that improved passive disease surveillance can be achieved by engaging communities in smallholder pig farming in Timor-Leste. Further research and testing of the approach in other districts and countries is recommended.

## Introduction

Smallholder pig production is extremely important in Timor-Leste with 74% of agricultural households raising an average of 3.4 pigs in 2019 (1). Pigs serve important cultural and financial roles in Timor-Leste households. They are consumed on special occasions, which can be sold for cash or exchanged ceremonially and their ownership fulfills social obligations (2). The national goal of increasing livestock numbers by 20% by 2020 reflected the desire in Timor-Leste to include smallholder pig farming in a strategy to improve food security and poverty alleviation (3). This recognizes the value of animal source protein in addressing the challenges of meeting nutritional needs, particularly for women and children (4, 5).

Improved pig production has been constrained by the unpredictable occurrence of disease and low productivity but remains a national priority (6). In Timor-Leste, native and crossbred pigs are typically produced with low inputs. Only one-third of pigs are kept fully confined, with semi-confined systems used for ~40% of herds and almost 30% free roaming (7). In each of these systems, there is a high burden of disease with excess piglet mortality, poor reproductive performance, and low body condition of sows with seasonal limitations in feed availability. Poor pig health and production outcomes reflect nutrition, which is predominantly based on feeding cooked household scraps, limited use of controlled breeding, very low rates of preventative parasite treatment (~10%), and limited engagement of veterinary expertise (7).

The low-input smallholder pig production systems in Timor-Leste proved to be vulnerable to the emergence of the African swine fever virus (ASFV) (8). This disease caused the death of more than 50,000 pigs during the initial outbreak in 2019 and has significantly disrupted the management of pigs in smallholder farms (9, 10). The continued spread of transboundary animal diseases threatens pig health in Timor-Leste. The movement of pigs across borders in the region is common (11). Surveillance for pig and other animal diseases is a national and international priority that requires an effective national veterinary service, as outlined by the World Organization for Animal Health (WOAH). The role of the veterinary service is also critical in supporting food security, agricultural and rural development, poverty alleviation, trade in animals and their products, and in environmental protection (12).

Extension activities for improved pig nutrition, farm management, and the use of bio-secure housing can be effective in supporting successful smallholder pig farming (9). The government veterinary technicians (para-veterinarians) are an important resource for Timor-Leste with the potential to provide widespread extension activities. However, an assessment of the national veterinary service using the WOAH Performance of Veterinary Services (PVS) pathway found inadequacies due to under-resourcing with an insufficient number of qualified personnel and a lack of facilities and equipment (13). Veterinary technicians provide the classical swine fever (CSFV) vaccination program for pigs and are responsible for responding to farmer reports of disease. There is an annual vaccination program against Classical Swine Fever, although vaccination rates can be low, especially in areas where animals are unpenned and hard to capture (13).

There is limited regular pig disease surveillance and a lack of pig husbandry extension for farmers (7, 14). Effective surveillance of pig diseases is more than a simple resourcing issue with the need for long-term effective partnerships built on trust between farmers and veterinary technicians (14, 15). Improved community engagement by veterinary technicians to facilitate disease reporting for surveillance can be achieved through pig husbandry training.

This article describes collaborative social and diagnostic research followed by a pilot community engagement program to improve farmer and technician knowledge, skills, and working relationships in relation to pig disease surveillance, biosecurity, and general husbandry. The methods and results are structured according to three distinct phases. Phase 1 was a qualitative research study to explore farmer and technician knowledge and experiences with pig diseases. The aim was to understand the barriers and opportunities for improved surveillance of pig diseases. Phase 2 involved community engagement training of technicians and implementation in 3 villages and a pilot disease investigation. The aim was to trial farmer learning to improve knowledge and skills and encourage disease reporting. Phase 3 was an evaluation of the community engagement program involving individual and group interviews with the aim of assessing outcomes and limitations. The discussion addresses the implications of the results in relation to improving pig disease surveillance, pig health, and pig production using community engagement methods.

## Methods

### Site selection

**Figure 1:**
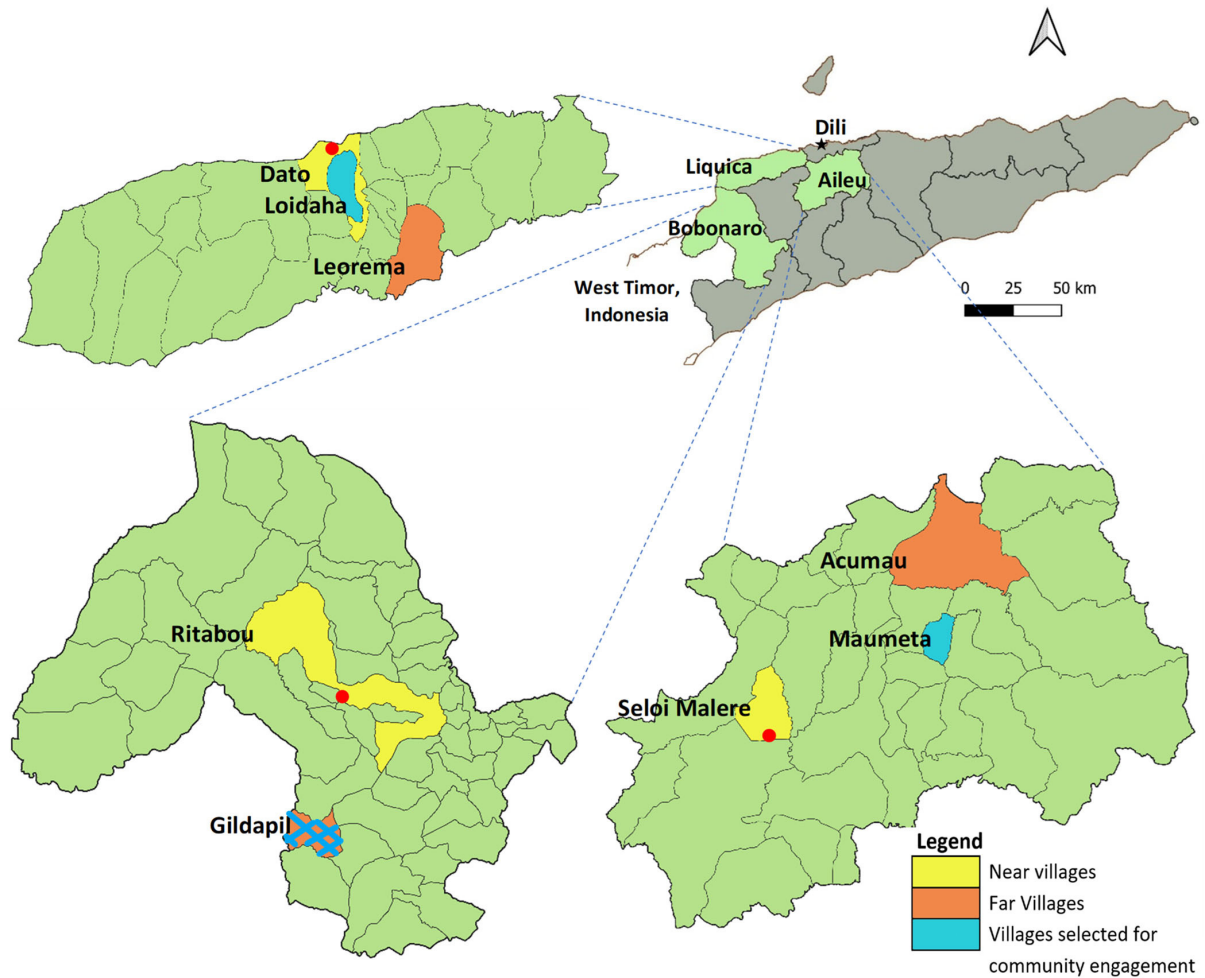
Location of study villages and engagement villages. The red dots are municipal towns.

Three municipalities (Liquisa, Aileu, and Bobonaro) were selected to participate in the program based on having significant pig populations and pig health issues (Figure 1). This information was obtained from national and district livestock databases in the Ministry of Agriculture and Fisheries.

For the Phase 1 qualitative study, two villages per municipality were selected (one close to and one remote from the municipal capital town). The aim was to explore any effect of distance on pig-raising methods, disease incidence, contact with MAF staff, access to animal health treatments, and information networks across each municipality. The villages selected were Ritabou and Gildapil (Bobonaro), Acumau and Seloi Malare (Aileu), and Dato and Leorama (Liquisa) (Figure 1).

For the Phase 2 community engagement pilot, technicians selected one village from each municipality based on the following selection criteria: (1) not involved with previous livestock extension activities, (2) remote from the municipal capital town, (3) high level of pig problems, and (4) willingness to learn and to work with MAF staff. The three selected villages were Gildapil (Bobonaro), Loidahar (Liquisa), and Maumeta (Aileu) (Figure 1).

Due to financial and logistic constraints, the disease investigation pilot was limited to the municipality of Bobonaro. Subsequent to the field surveillance and case investigation workshop, the technicians in Bobonaro encouraged farmers across the municipality to report pig diseases. They undertook disease investigations when alerted of grower and adult pig deaths.

### Pig disease knowledge and reporting study methods

Phase 1 qualitative study was conducted in December 2020 to explore the experiences and knowledge of pig farmers, village leaders, and veterinary technicians on pig diseases and reporting, treatment methods, and access to information or assistance. Semi-structured interviews were conducted with six village leaders (five men, one woman) and 16 veterinary technicians (11 men, 5 women). Two focus group discussions (FDGs) were held in each village (one for women, one for men), totaling six FGDs with 133 farmers (71 women, 62 men). All interviews and FGDs were conducted in Tetun (the national language of Timor-Leste) and audio recorded.

Interviews with village leaders were held at their homes. The interview guide included 20 open-ended questions related to village pig-keeping practices and disease reporting by farmers. It also asked about the frequency of and reasons for MAF visits and the potential for the collection of samples from pigs. The impact of any ASF outbreak on the village was asked along with information sources and needs. Interviews with veterinary technicians were held at the municipal livestock offices. There were 37 predominately open-ended questions relating to their experience with raising pigs, challenges to working with pig owners, frequency of village visits, and farmer reporting of sick/dead pigs. It also covered pig disease identification and treatment, confidence in taking samples and doing post-mortems, and suggestions on how to improve animal health services.

For the focus group discussions, the village leader was requested to provide a list of farmers that met the following criteria: (1) pig-raising experience, (2) have at least a primary level of education to participate in numerical exercises, (3) at least 25 years of age, and (4) not holding leadership role or a person with particular influence in the community. From this list, participants were randomly selected by gender and invited to join an FGD with the aim to have 12 participants per focus group. However, some participants in every FGD had no schooling and were younger than 25 years. One village invited more than 12 participants. In Timorese culture, it is often difficult to refuse participation so criteria were not strictly followed. Each FGD was held at a community venue with a duration of ~2 h and audio recorded.

**Figure 2:**
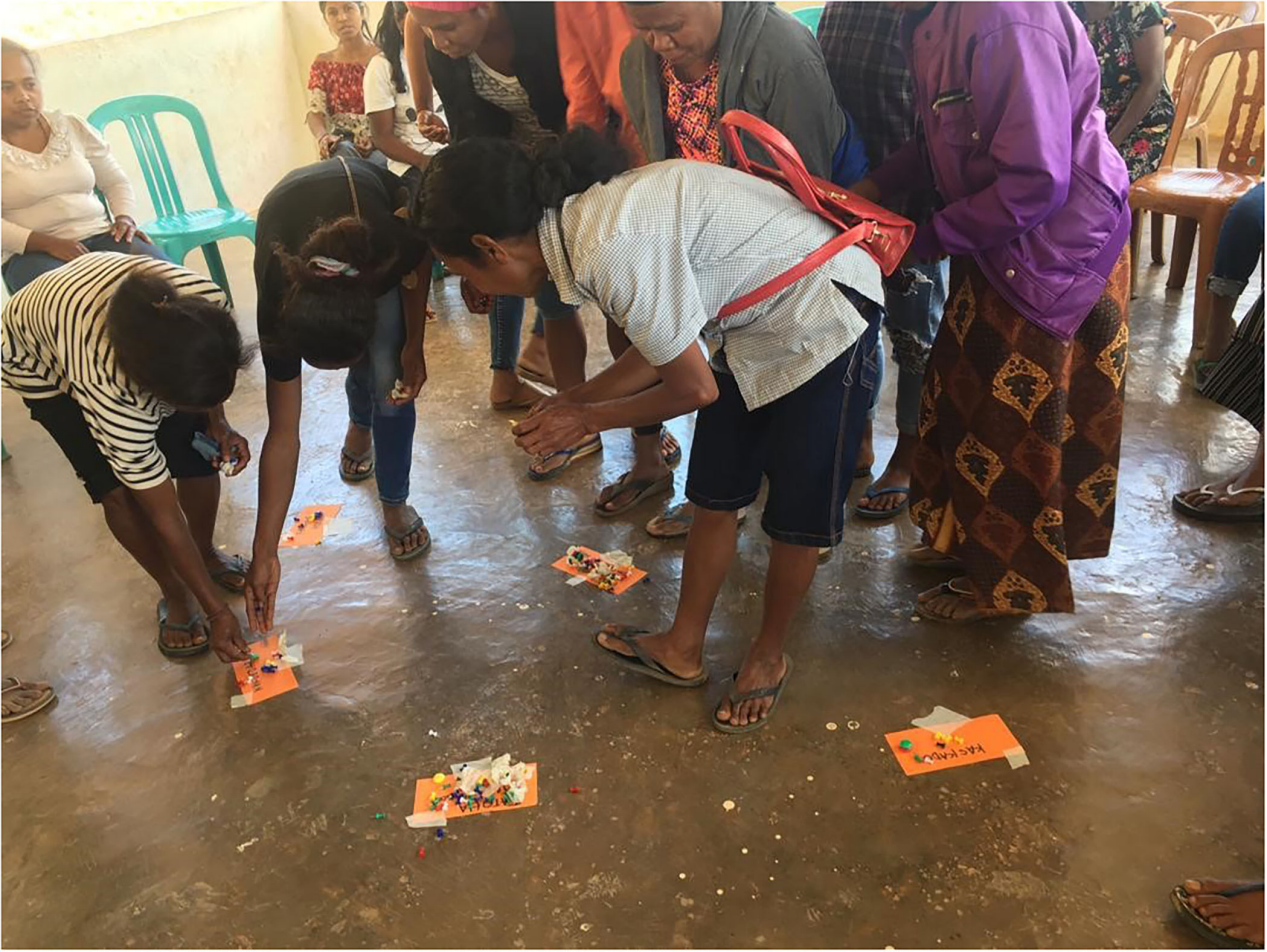
Women's focus group doing disease ranking exercise at Gildapil village.

Basic demographic information was obtained through a short interview with each participant held prior to the start or at the end of the group discussion. The FGD had five sections related to (1) benefits and challenges of pig raising, (2) pig disease identification, severity ranking, and treatments, (3) impacts of ASF, (4) reporting to MAF and level of satisfaction with MAF services, and (5) information sources and delivery preferences. Participants were asked one by one about issues that were drawn up on tables on a whiteboard. For disease severity rankings, each farmer was given 10 pins and allocated the pins according to the severity of each disease (Figure 2).

Interview data were entered into Excel spreadsheets by the Timorese team in English and analyzed to collate answers to each question. FGD data were compiled into separate reports in Tetun, translated to English, and analyzed using Microsoft Word using themes. Quotes were obtained from the audio recordings. Analysis was reviewed by the Timorese team.

### Community engagement training and implementation methods

For Phase 2, 31 veterinary technicians were trained in community engagement and diagnostic investigation of pig diseases in each municipality in July 2021. Topics, activities, and discussions covered at each workshop included: (1) understanding community engagement (its aims and methods), (2) previous experiences with community engagement, (3) a village analysis exercise, (4) design of a best practice village CE program, and (5) how to monitor and evaluate CE outcomes. During workshops, the technicians identified one village from each district for maximum impact in improved management of pig health and production. Each team designed and implemented a structured farmer learning program to motivate farmers and build knowledge on pig diseases, biosecurity, and husbandry.

The farmer learning program was conducted over 6 weeks for 15 self-selected farmers per village (a total of 45 households across three districts) in November–December 2021. Sessions included: (1) general introduction, (2) pig nutrition and feed formulation, (3) sow, piglet, and boar management, (4) disease identification and treatment, and (5) biosecurity protocols including building and cleaning secure pens, and how to report sick or dead pigs. Four technicians from each team were then visited every 1–2 weeks for the next 4 months to motivate farmers and monitor practice changes.

### Disease investigation training and implementation

A 5-day field surveillance and case investigation workshop were held in Dili in February 2021 for 28 veterinary technicians (13 Bobonaro, 8 Liquisa, and 7 Aileu) (6 women and 22 men). The workshop provided instruction on responding to reports of pig disease for optimal diagnostic outcomes. It involved presentations, discussion sessions, role-play activities, and hands-on practical sessions and covered the design and completion of case investigation record forms and the collection and preservation of pig diagnostic specimens from live pigs and at post-mortem. From April 2021, all veterinary technicians in each municipality were provided with equipment (record form, post-mortem kit, sample collection pots, PPE, and disinfectant) and fuel for transport to attend farm to collect data and samples on farmer reports of pig disease fitting the case definition (mortality of grower or adult pig without an obvious non-infectious cause).

During the on-farm investigation, details about the affected pig, other pigs on the farm, and the farm management were recorded, and a post-mortem was performed on dead case pigs to collect a broad selection of tissue samples that were preserved in 10% neutral buffered formalin and 80% ethanol. These preserved samples were transported to the National Veterinary Laboratory in Dili and on to the Berrimah Veterinary Laboratory in Darwin, Northern Territory for histopathology examination and molecular testing. The available budget imposed a limit of 27 cases for laboratory diagnostic investigation.

Formalin-fixed tissues were prepared for histopathology according to standard methods, stained with hematoxylin and eosin, and examined by a veterinary pathologist. The following tissues were examined from each case: heart, lung, spleen, kidney, liver, jejunum, and tonsil. Ethanol-fixed tissues from each case were prepared for molecular tests for ASFV, CSFV, and *Pasteurella multocida* for all cases, and other pathogens if indicated by the case history and histopathology. A 0.2-g portion of each tissue sample, spleen for ASFV and CSFV, and lung for *P. multocida*, from each pig, were added to phosphate-buffered gelatin saline (PBGS) solution and homogenized with 0.3 g of 0.5-mm Zirconia/Silica Beads (TissueLyser II, Qiagen) prior to extraction of total nucleic acids using a magnetic bead–based extraction kit (MagMax CORE Nucleic acid purification kit, Thermo Fisher Scientific, Austin, TX) according to the manufacturer's instructions, using a magnetic particle handling system (KingFisher 96, Thermo Fisher).

The real-time polymerase chain reaction (qPCR) assays for ASFV and CSFV were conducted according to the method described by Haines et al. (16). Molecular detection of *P. multocida* used a modification of the assay described by Corney et al. (17). In each case, the reactions were prepared using a commercial master mix (AgPath-ID one-step RT-PCR kit, Thermo Fisher) with 5 μl of purified nucleic acid in a total reaction volume of 25 μl. The assays were run on a QuantStudio 5 real-time PCR machine (Thermo Fisher) in normal mode, under the cycling conditions specified by the manufacturer for the Ag-Path master mix. The fluorescence threshold was set manually at 0.05, and the background was automatically adjusted. Results of real-time qPCR assays were expressed as cycle threshold (Ct) values when positive or classified as negative if amplification was observed after 45 cycles. Positive and negative control samples were included on each plate for quality control according to operating procedures compatible with ISO17025 accreditation.

### Evaluation methods

Phase 3 evaluation of the impacts of the community engagement and farmer learning program was conducted in March 2022. Semi-structured interviews were held with 27 farmer participants (18 women, 9 men) who had participated in the farmer training and were available to be interviewed. The farmer interview consisted of 17 questions related to the most useful sessions, estimated changes in knowledge gained from the learning program, and changes in pig management practices since the program. Challenges and future plans for pig management were also asked along with their motivation to report sick and dead pigs to technicians and desired assistance from MAF for pig raising. Twelve technicians (11 men and 1 woman) who were involved in farmer training and follow-up visits were interviewed in groups. Technicians were asked about the positive and negative aspects of the farmer training and follow-up visits for farmers and themselves. They rated changes in their knowledge and skills in each pig husbandry topic. They also rated changes in confidence and motivation levels to work with communities and farmers. Interviews were audio recorded, and data were entered into Excel. Data were analyzed using descriptive statistics and compiling qualitative responses into themes.

### Ethics approval

Ethics approval was obtained from the University of Sydney Human Research Ethics Committee (Project number 2020/122). An information sheet was provided and verbally explained to all participants for each interview or focus group discussion. Informed consent was obtained from participants through either a written signature or thumbprint. Participants were able to decline to participate or withdraw from the study at any time.

## Results

### Farmer knowledge and experience with pig diseases and reporting

The following Phase 1 results are from the six focus groups involving 133 people. Background information on gender, ages, and education levels is shown in Table 1. Ages varied from 17 to 71 years. The more remote villages in Aileu and Bobonaro had a greater proportion of people with no schooling or primary education only. Liquica farmers had higher education levels, probably due to proximity to Dili and shorter distances between towns. Most farmers were middle aged with Dato having the youngest group. The average number of pigs per household was 2.0. Dato had the highest average pig number (2.9), and Gildapil had the lowest (0.85).

**Table 1:** Gender, ages and education of farmer respondents.

**Village**	**Men**	**Women**	**Age range (years)**	**Mean age (years)**	**Education level**
Leorama	7	5	21–61	39.5	3% no school
Dato	6	8	17–40	25.6	8% primary school
					81% secondary school
					8% post-secondary
Acomau	18	20	19–71	43	42% no school
Seloi Marlare	11	9	24–59	42	31% primary school
					21% secondary school
					5% post-secondary
Gildapil	9	11	19–52	34	27% no school
Ritabou	11	17	25–68	44	33% primary school
					27% secondary school
					12% post-secondary

### Recognition and severity rating of pig disease signs and pig deaths

Farmers were unable to name exactly which diseases were affecting their pigs, but they were able to describe signs. Their descriptions fell into two main groups of signs; ones related to viruses (CSF and ASF) and parasites (external and internal). Most groups talked about “Tatoha” which refers to hypersalivation, difficult breathing, and coughing. Body changes such as red spots, swollen head, fever, lameness/lying down all the time, and loss of appetite were often described in addition to “Tatoha.” Less severe signs related to parasites (itching, diarrhea, and weight loss) as farmers said it did not kill pigs and occurred over a longer timeframe. Women mentioned a broader range of signs than men as they are often the primary carers of pigs (Table 2). Participants were then asked to rank the five most severe signs by allocating 10 pins (Table 2).

**Table 2:** Ranking of disease signs for severity by allocation of 10 pins.

**Disease**	**Leorema**		**Dato**		**Acumau**		**Seloi M**		**Gildapil**		**Ritabou**		**Total (rank)**
**(W-Women, M-men)**	**W**	**M**	**W**	**M**	**W**	**M**	**W**	**M**	**W**	**M**	**W**	**M**	
Distress, loss of appetite then pigs die	30												30
Trembling	11				20							5	36 (10)
Fever/fever and redness	10	6	6	33	18			24				13	110 (3)
Vomit	6		4	3									13
Appetite loss		11	6		27	17				12		5	78 (7)
Hard breathing and hypersalivation	1	30	16	6	27	24	20	25	17	21	10	5	202 (2)
Coughing	1	12	10	6	10	17			17		10		93 (4)
Itchy		5					19	21		19	19	6	89 (6)
Lose weight		1		14									15
Worm in lung		1											1
Sudden death			79		18	58	48	25			24	58	310 (1)
Constant lying down/lameness			6	10	19				40		24		89 (6)
Scabies					9								9
Swollen body/head					18	17			35	21			91 (5)
Diarrhea					21					16			37 (9)
Parasite					30		3				3	9	45 (8)

Pig death estimations from group participants in the last year (2019–2020) totaled 328 pigs affecting all pig age groups (Table 3). Signs reported were similar across all districts. The disease signs that occurred after the ASF outbreaks in 2019 were more acute with groups reporting sudden death or death after showing signs for only 1–2 days. Several groups commented that the disease spread quickly. Gildapil groups reported fewer deaths during this period which possibly linked to farmers claim that they had a village law that dictates all pigs are confined with no outside pig meat allowed into the village.

**Table 3:** Pig deaths in the last year (2019–2020).

	**Leorema**	**Dato**	**Acumau**	**Seloi Malare**	**Gildapil**	**Ritabou**	**Total**
Sows/gilts	12	15	20	18	6	29	100
Boars	4	8	13	19	2	25	71
Young pigs	15	3	34	21	6	78	157
Total	31	26	67	58	14	132	328

### Treatment methods and reporting to MAF

Group respondents were asked about treatments they used to treat the listed disease signs. All groups said they use traditional medicines. Traditional and modern treatments were deemed more successful for external parasite infections than viruses. Some groups described the process of trying to get help with sick pigs. Table 4 shows how farmers treated diseases and their views on the effectiveness of treatments and assistance. Only one group mentioned prevention strategies (Leorema men).

**Table 4:** Prevention and treatments actions by farmers.

**Diseases**	**Prevention and treatment (group name)**
Sick pigs in general	Confine, clean/spray pigs and treat with traditional medicine (some work/pigs recover, some don't work/pigs die). (Dato women and men).
	No modern medicine as no shop nearby. Field technicians come and treat but pigs continue to get sick, lose weight and die. We understand a bit of theory but don't know proper medicine to treat (Dato men)
	Use traditional medicines or can buy medicines at agricultural shops and giving injection by ourselves. Because when calling the technicians they ask for petrol to fill their motor bike (Ritabou men)
Tatoha (coughing, difficult breathing)	Give traditional medicines “aeftata” = wood charcoal. Scrape then mix up with water and pigs drink. (Acumau men)
	Burn crowbar (till hot) and put in the food then feed the pig. Buy medicine “amoxicilin, cotrimox at the small shop (crush them then put in the water drink or food) Slicing the tree bark then boil and give it to pig. (Acumau women)
	Offering sugar and water—sometimes pigs recover. Traditional medicine mixed up with water (Seloi M women and Ritabou women)
	Buying medicine in small shop—ampicillin (Gildapil women)
Parasites (worms)	Traditional medicine: “aeftata”—mix charcoal with salt water and give to pigs to drink (Acumau men)
	Just buying the medicines nearby (Seloi M women and Ritabou women)
	Using traditional medicines we get a good result. Also can use commercial medicine that sells at agricultural shop (all treatments we did have a good result). All medicines we treat by ourselves (Ritabou men)
Swollen head and suddenly dead	Traditional medicine- soak cattle and monkey leather and give to drink (Acumau men)
	Called field technician (Seloi M men). Some treated survived, some dead.
	Tried traditional medicine but still die (Gildapil men)
Inflamed liver, swollen head, hard breathing, and hypersalivation, red spots, lameness. Sudden death. [ASF]	Tried many medicines but did not work (Acumau men and women; Leorema men)
	Tried using tree bark but did not work (Leorema women)
	Scared to eat pork or bring pork from outside (Leorema men)
	Cannot do anything (Seloi M women and men, Gildapil men and Ritabou women and men)
Itching/Scabies	Clean with detergent and rub with oil. Rub with kerosene has a good result as all the ticks die and make hair shiny. Some people ask field techs to give them assistance (Acumau women)
	Mix up water and detergent then pour on whole body. The result was sometimes recovery but sometimes die. Another traditional medicine is kerosene mixed up with bitter flower then rub on the body (Seloi M women and Ritabou women)
	Using traditional medicine/herbal “tabacco,” detergent and kerosene then pouring on the body (Gildapil men)
	Use traditional way like pouring oil and herbal leaves (Seloi M men)
Diarrhea	Informing field tech to doing treatment even though we don't know exactly the drugs (Gildapil men)

There was a very low level of reporting to MAF (only 18.8% or 25/133). Reasons given related to unfamiliarity with technicians, distance to the MAF office, lack of trust that anyone will visit, and pigs dying anyway. For the few farmers that did report to MAF, the reasons were to get treatment for sick pigs and get advice about disease prevention. Most groups were not satisfied with the frequency or quality of animal health services. All groups said they wanted animal health services to improve. They needed information on how to raise pigs, prevent diseases, and treat sick pigs. Preferred communication was village visits, community meetings, scheduled programs, mass media, and the use of posters and pictures. Most farmers agreed with diagnostic sampling and post-mortems if it meant knowing what was wrong with their pigs and how to prevent further illness or death. However, there was some reluctance to euthanize pigs unless the reasons are clearly explained to them, and there is no hope of other treatments working.

### Veterinary technician knowledge and experience of pig diseases and reporting

The veterinary technicians interviewed were eleven men and five women. Ages ranged from 27 to 48 years (average 32 years). Four technicians had secondary school education and twelve had post-secondary education. Length of time as a veterinary technician ranged from 4 to 17 years with an average of 13 years.

### Recognition of pig diseases, diagnosis, sampling, and treatment

Most technicians (80%) could identify lice, parasites, diarrhea, and scabies signs but only two people said they could identify screwworm flies. About half of the technicians said they recognized CSF and half said ASF. Although all technicians could describe disease signs, some suggested treatments that were inconsistent with the disease. For example, antibiotics were suggested for viruses and parasites by 10 technicians, which would only treat secondary bacterial infections. Most technicians had no or little experience with sampling or post-mortems. A few older staff had more experience which was reflected in higher confidence levels. Staff were more familiar with taking fecal samples than blood or skin. Only one person was very confident with post-mortems.

### Challenges working with farmers and reporting diseases

Challenges raised by technicians related to farmers not trusting or accepting them for the same reasons given by farmers. The following quote summarizes some of the practical challenges faced by technicians working with farmers:

“*Sometimes I went to the location but when I arrived the farmers were going to doing another activities, sometimes they report that their animal were sick but when I arrived their animals are free roaming so hard to treat. Distance of location sometimes difficult to reach. No freezer to storage the vaccine so I have to go to Aileu town and get the vaccine and go to field. No operational support (fuel and per diem).”* Aileu technician (male).

The main reasons farmers tend not to report sick or dead pigs to MAF according to technicians were farmers' lack of disease knowledge, fear of being charged fees, and simply not knowing who to contact. The following quote also highlights the systemic problem with lack of diagnosis results back to technicians and farmers:

“*Many times diseases team come to the field and collect samples then carried to Lab for testing but they never confirm back the result to the field techs and animal owner so it makes farmers lose trust in reporting cases to field techs.”*

### How to improve disease surveillance, reporting, and information to farmers

Veterinary technicians' suggestions on how the diagnostic system could be improved included (1) making sure results were returned to technicians and farmers, (2) providing necessary equipment and training for collecting samples, (3) increasing the number of technicians, and (4) providing better operational support. Most technicians said that farmers needed information on animal husbandry, animal health, how to raise pigs, and how to prevent diseases and control diseases. Four technicians suggested that it would be easier and more effective if they could provide information and assistance to small groups of pig owners. Several technicians said that regular meetings with farmers were needed and good coordination between technicians and farmers on how to raise pigs using simple technical language. The following quote summarizes some of the suggestions:

“*Strengthen good relationships with farmers, all the animals should be confined so it would help us to control, and continuous public awareness regarding diseases and animal husbandry systems.”*

### Disease investigation results

The 27 cases of pig mortality were predominantly from farms with local or local-crossbred pigs, with less than seven pigs in total, and adult or growing pigs were affected rather than piglets. Most farms were confining the pigs and providing a diet derived from kitchen scraps. The diseases that were seen were of short duration (median 4 days) and only four involved weight loss. There was a broad range of clinical signs with fever and lost appetite being most frequently observed (Table 5). ASFV was detected by qPCR in 70% of the cases, while there was no evidence of CSFV. Tests for *P. multocida* were positive in 33% of the cases, although this bacteria is part of the normal flora of pigs, it is capable of causing a significant potentially fatal disease, especially when other factors compromise the pig's health (18).

**Table 5:** Clinical signs, necropsy, and histopathology of 27 reported cases of pig mortality with suspected infectious disease etiology in 2021 in Bobonaro municipality.

**Clinical signs**	**Present (%)**	**Absent (%)**
Lost appetite	81	19
Fever	63	27
Respiratory effort increased	48	52
Skin hemorrhage	30	70
Recumbency	19	81
Weight loss	15	85
Lameness	15	85
**Necropsy observations**		
Lesions observed in viscera	81	19
Parasites observed	37	63
**Histopathology lesions**		
Lesions consistent with ASF (spleen)	63	37
Evidence of kidney disease	44	56
Evidence of parasites (liver)	78	22
Lung pathology, predominantly parasitic	81	19

Notably, all but one of the *P. multocida* positive pigs were also positive for ASFV, suggesting secondary bacterial colonization of the lung following debilitation due to ASFV infection may have occurred. Secondary involvement of *P. multocida* in the pathogenesis of ASF has been noted previously due to the immunosuppressive nature of ASFV infection (19). In regions free from the virus, ASFV introduction manifests as a severe peracute, hemorrhagic viral infection of susceptible naive pigs. The disease is characterized by marked pyrexia, cutaneous hyperemia, and sudden death with morbidity and mortality approaching 100%. As ASFV establishes in a region, more chronic forms of the disease become increasingly evident, and this is characterized by recurring pyrexia, abortion, emaciation, growth retardation, and other non-specific findings (19).

Histopathology identified lesions consistent with acute to subacute ASF in each of the cases that tested positive by qPCR, confirming that this pathogen was a significant contributor to the cause of death and largely consistent with the reported clinical signs. Histological evidence of ASFV infection included extensive necrosis of mononuclear phagocytic cells throughout lymphoid tissues, degeneration of renal tubules with cast formation, necrosis of periportal hepatocytes with infiltration of lymphocytes through portal regions of the liver, and degeneration of vascular endothelium and fibrinoid arterial change in various tissues. Splenic changes were the most obvious and consistent finding in the sampled pigs.

In the eight cases of pig death that were not associated with ASFV, the suspected disease etiologies were bacterial and parasitic (verminous). Aside from the histopathological evidence of ASFV infection, 75% of pigs in all cases showed histopathological evidence of pneumonia. The majority of these were graded as being either moderate (13/27 cases) or severe (7/27 cases). This pathology was considered to have contributed significantly to the reported morbidity. Four of the cases that were not associated with ASFV had significant pulmonary lesions, and three of these were determined to be due to intralesional parasites. Pig lungworm (*Metastrongylus* sp.) was either observed in the pulmonary lesions or suspected given the pathology present. At least one pig demonstrated evidence of severe bacterial bronchopneumonia with subsequent bacterial septicemia disseminated necrosis in several tissues. However, this lung sample returned negative PCR results for *P. multocida*, suggesting an alternative bacterial etiology was responsible. Of the remaining four cases, three had mild to moderate hepatitis, likely due to parasitic migration through the liver, and one had no microscopic pathology noted. Antimicrobial resistance in pigs is not an issue in Timor-Leste as there is very low use of antibiotics as found by Ting et al. (20).

Histopathological examination identified other disease processes that were likely to be present as production-limiting issues in other pigs at the farms, and which are amenable to improved preventative health care. For example, the evidence of infection with pig lungworm (*Metastrongylus* sp.) was found in 18/27 sampled pigs, with intralesional parasites observed in five of these. This nematode typically causes heavy infections in younger animals and can contribute to ill-thrift and secondary bacterial pneumonia in adult pigs, especially those with high-exposure to earthworms, the intermediate host of the parasite (21).

### Evaluation of the pilot community engagement program

#### Farmer interviews

Twenty-seven of the 45 households (60%) who participated in the farmer learning program were available for interview in March 2022. Table 6 shows their gender (18 women, 9 men), age ranges, number of pigs at the time of the interview, and how many of them confined their pigs in pens. Participants in Loidahar had the highest number of pigs, and Gildapil had the lowest. Two-thirds of those interviewed said they were confining their pigs either in pens or tethered.

**Table 6:** Gender, age, and pig ownership of 27 farmers that participated in the evaluation interviews.

**Village municipality**	**Maumeta, Aileu**	**Loidahar, Liquica**	**Gildapil, Bobanaro**	**Totals**
Respondents	9	10	8	27
Genders	5 F, 4 M	7 F, 3 M	6 F, 2 M	18 F, 9 M
Ages (years)	21–75	18–60	19–26	19–75
Number of pigs	1–6	1–14	1–2	1–14
Confinement of pigs	5 farmers	9 farmers	8 farmers	22 (75%)

F, female, M, male.

#### Most useful training sessions

Farmers thought that the feeding and biosecurity training was most useful (Table 7). Breeding and health management were seen as less useful possibly because farmers know how to breed pigs already. Feed formulation using uncooked feedstuffs and building biosecure pens were new practices for these farmers.

**Table 7:** Most useful training sessions (number of responses) (*N* = 27).

	**Feeding**	**Biosecurity/pen construction**	**Health management**	**Breeding**	**All topics useful**
Maumeta	6	3	1	0	1
Loidahar	4	6	1	1	2
Gildalpil	7	5	0	0	0
Total	17	14	2	1	3

#### Knowledge and practice change

Farmers were asked to self-rate how much their knowledge had increased following the training sessions for each topic. Table 8 shows that Maumeta farmers had a small knowledge increase. Loidaha and Gildapil farmers thought their knowledge had moderate increases across all topics with a particular interest in disease management. Eighteen of the 27 farmers interviewed (66%) said they had changed feeding practices. Changing to dry feed mixes using locally available ingredients appealed to many farmers as it reduces labor and avoids contamination. Only seven households interviewed had built pens (26%) due to the prohibitive cost of materials. However, additional households are keen to build biosecure pens if they can afford them or be subsided in the future. The main challenges to continuing biosecure practices were cited as: the cost of building pens (16 responses), finding feed (6 responses), lack of time (55 responses), and pigs getting sick (2 responses). Farmers need a longer period to practice these skills and become confident that they can help reduce disease incidence and lift the productivity of their herds.

**Table 8:** Self-reported change in knowledge from farmer training (*N* = 27) (mean values).

**Knowledge change**	**Maumeta Aileu (*n* = 9)**	**Loidahar Liquisa (*n* = 10)**	**Gildapil Bobanaro (*n* = 8)**
Feeding	2.6	2.7	2.9
Breeding	2.0	2.6	2.7
Diseases and health management	2.0	2.8	3.4
Biosecurity	1.0	2.8	2.9
Pen construction	2.3	2.9	2.6

1 = no change, 2 = small increase, 3 = moderate increase, 4 = high increase, 5 = very high increase.

#### Changes in farmer motivation to report sick or dead pigs to MAF

**Figure 3:**
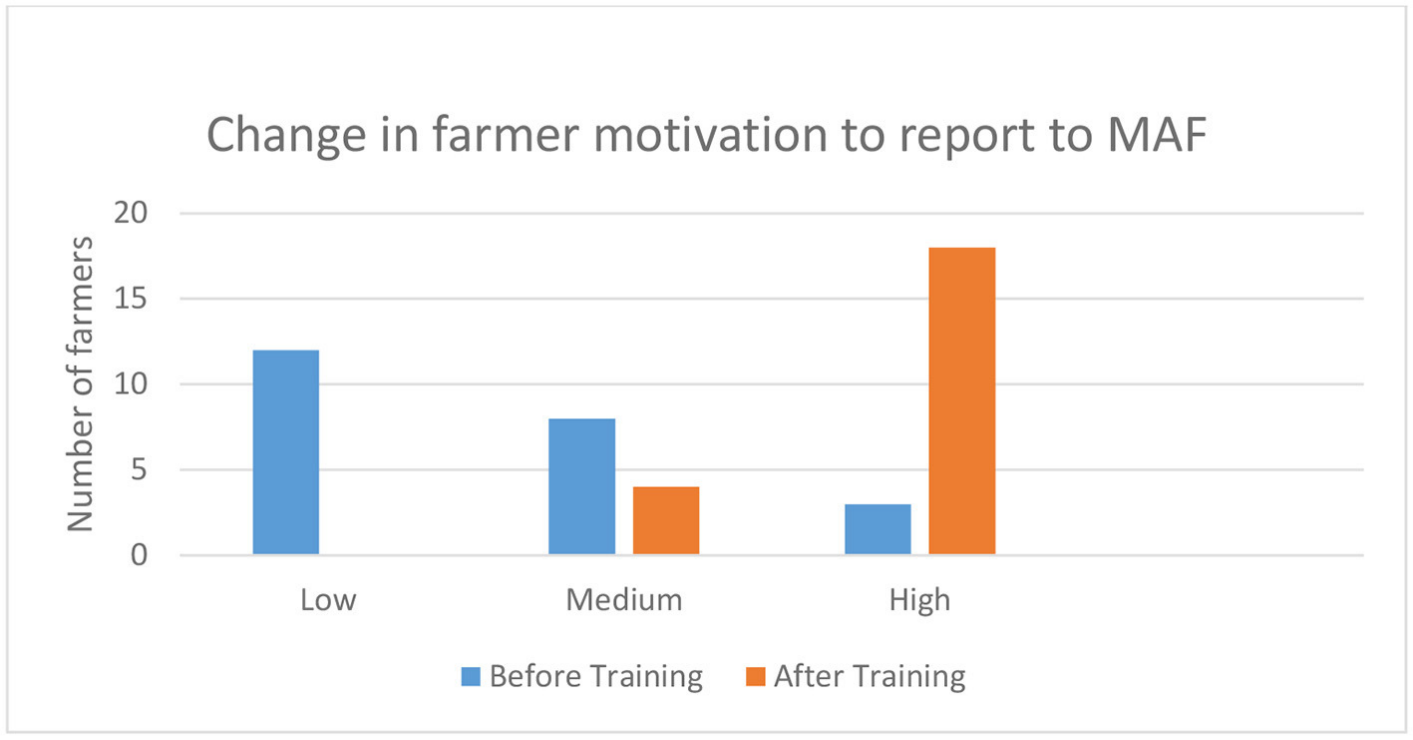
Self-reported change in farmer motivation to report pig diseases to MAF (*N* = 27) (mean values).

Farmer motivation to report to MAF increased substantially as a result of learning about the importance of good disease management (Figure 3). The reasons for being more motivated to report were: to prevent the spread of diseases (six responses), gain knowledge from training (three responses), obtain information from the technician in Aileu (five responses), and realize there is a need to report (one response).

#### Future plans and assistance needed

Thirteen respondents (48%) wanted to build new pens for confining pigs, nine respondents (33%) wanted to buy more pigs and five respondents (18.5%) had no plans. All farmers wanted more regular training on pig husbandry, especially diseases, materials for making pens, treatments for pigs, additional training on feed formulation, and to be provided with pigs.

#### Technician interviews

Twelve veterinary and livestock technicians (11 men and one woman), who were mostly involved in the farmer training program (three Aileu, four Liquisa, and five Bobanaro), were interviewed at their respective municipal offices. Ages ranged from 32 to 52 years with most in their early 30 s.

### Positive aspects of farmer training and follow-up visits

The positive aspects of the farmer training mentioned by Bobonaro technicians were that farmers now know how pigs get sick due to feeding kitchen scraps or people who carry diseases into their pens, so they understand the need for biosecurity. Aileu technicians said that farmers learned how to change from a traditional to a more modern pig-raising system by building pens, preventing disease, knowing when females are ready to mate, how to formulate and prepare uncooked food, and which treatment to use for different diseases. The advantages of follow-up visits mentioned by the Aileu group were “getting to know farmers well so they understand the role of MAF staff, able to reinforce message about sanitation, farmer slowly learns about disinfecting and cleaning, farmers can explain why they cannot adopt some practices due to lack of money and materials.” The Liquisa group said the follow-up visits enabled them to see farmers starting to confine their pigs using local materials, prepare food with available local ingredients including using silage, and an increase in pig numbers. Similarly, the Bobonaro group observed farmer practices such as preparing silage and using boots when entering the pen and disinfecting pens. Farmers from a neighboring village approached them about delivering the same learning program for their village.

### Negative aspects of farmer training and follow-up visits

Negative aspects of farmer training mentioned included many farmers not applying learning from the training as they still prefer to use traditional medicines and methods (Aileu). Some farmers in Loidahar assumed that the project would offer pigs and materials for pen construction to all people so some social jealousy emerged (Liquisa). Similarly, Bobanaro technicians reported that farmers thought that all pig food will be given by MAF technicians so some farmers were not motivated to attend. A common issue was different family members attended different sessions due to their time limitations.

Negative aspects of follow-up visits observed by technicians were that some farmers found regular visits intimidating as if they were being coerced to build pens and adopt new feeding practices (Aileu). The Liquisa group felt somewhat disillusioned because farmers still prefer to use traditional methods and are not motivated to report sick pigs. The Bobonaro group observed that farmers were unable to buy nipple drinkers in the town so they did not install them.

Technicians made many suggestions for improving community engagement and farmer learning in the future. They felt more training on feed formulation and housing management was needed, more assistance to build biosecure pens and source pigs, run cross visits between villages for farmers and technicians to expand their learning, and extend the program to other villages and local authorities. However, to do this, more staff are needed to cover more villages.

### Knowledge and skill change

**Figure 4:**
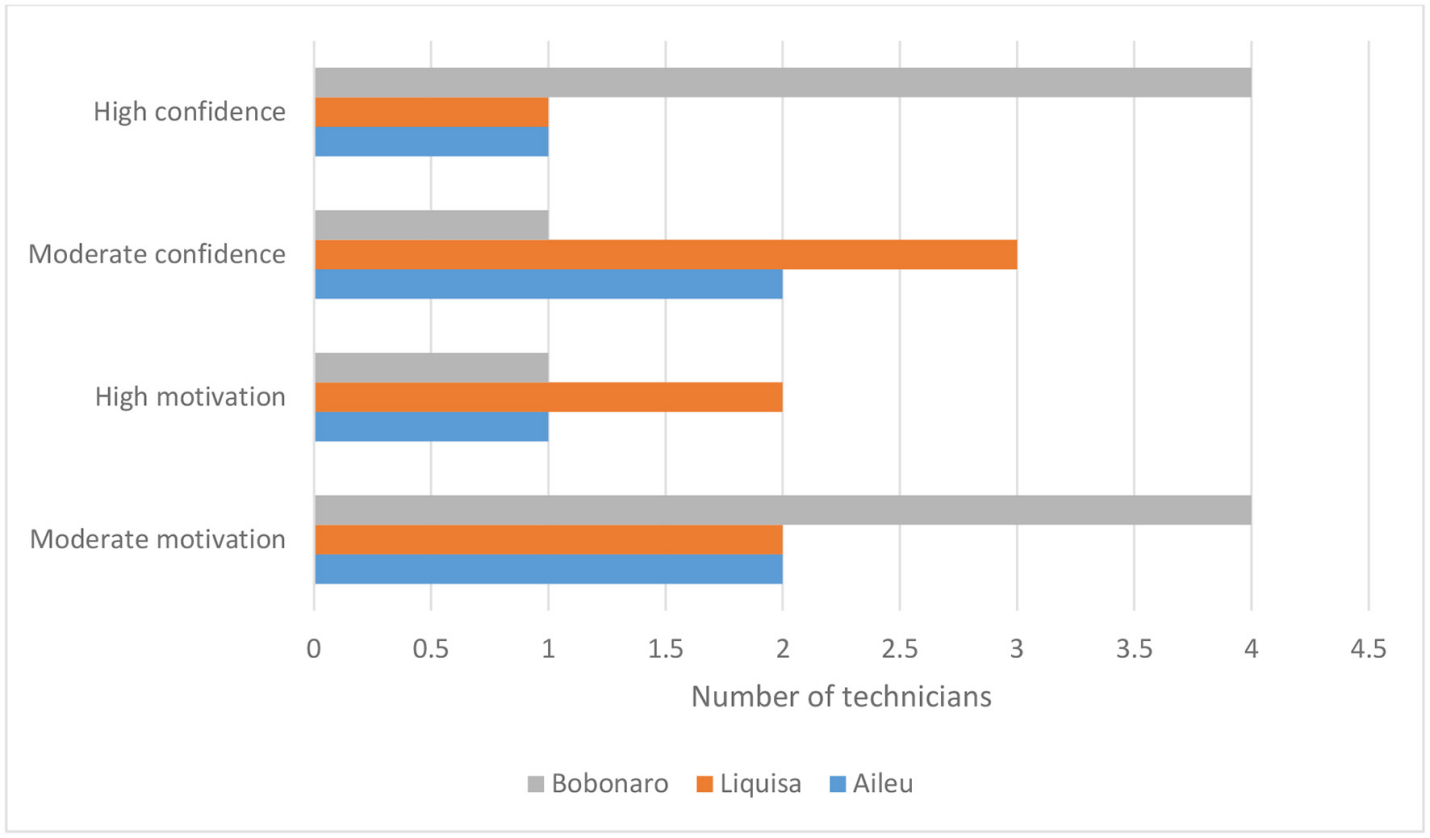
Self-reported change in technician motivation and confidence to work with farmers (*N* = 12).

There was wide variation in rated knowledge and skill change among technicians. Bobanaro staff had the highest increase, especially for learning how to work closely with communities and facilitate farmer learning. Liquisa staff ratings were mostly small to moderate and Aileu staff had the lowest scores except for feeding knowledge (Table 9). All staff reported increased motivation and confidence to work with farmers (Figure 4).

**Table 9:** Self-reported changes in knowledge and skills of technicians (*N* = 12) (mean values).

**Knowledge change**	**Aileu (*n* = 3)**	**Liquisa (*n* = 4)**	**Bobanaro (*n* = 5)**
Feeding	4.3	2.1	3.8
Breeding	1.6	3.0	4.2
Diseases and health management	2.4	3.2	4.1
Biosecurity	2.0	2.2	4.2
Pen construction	3.0	2.2	3.8
How to work more closely with communities	2.7	2.5	4.4
How to facilitate farmer learning	3.0	2.5	4.5
What samples to collect from a sick or dead pig for disease investigation	3.0	3.0	4.0
**Skills change**	**Aileu**	**Liquisa**	**Bobanaro**
Mixing feeds and how to feed	2.5	2.5	3.6
Boar and sow management	2.0	3.0	4.3
Diagnosing diseases	2.7	3.0	4.2
Biosecurity procedures	2.7	2.7	4.8
Pen construction	2.7	2.7	4.2
How to collect fecal sample	3.0	3.0	4.2
How to collect blood sample	2.3	2.5	3.8
How to do post-mortem	2.0	2.5	4.4

1 = no change, 2 = small increase, 3 = moderate increase, 4 = high increase, 5 = very high increase.

## Discussion and conclusion

The qualitative study of farmer experiences with and knowledge of pig diseases confirmed significant mortalities from ASF and possibly CSF since 2019. Although farmers could not name specific diseases, most respondents recognized signs of viral and parasitic infections, and they used a combination of traditional and modern treatments if available.

Smallholder farmers in developing countries such as Timor-Leste with chronic poverty and lack of veterinary services, often experience high pig mortalities from diseases, particularly during major outbreaks (11, 22–24). Low investment in pig farming leads to low biosecurity, which in turn leads to higher disease risk (9, 24, 25). Once pigs are affected, household income and confidence decrease, making it more difficult to implement preventative and control measures (23, 26). In this context, public and private veterinary technicians are crucial for providing effective disease surveillance (active and passive), while also assisting farmers to improve pig health and husbandry strategies (12, 27).

However, the qualitative study revealed limited reporting of sick or dead pigs by farmers to veterinary technicians due to a lack of trust in the veterinary diagnostic system as also reported by Ting et al. (20) and Hunter et al. (2). Although farmers reported occasional active surveillance visits by scientists, there was no feedback or information about what diseases were found or how communities could prevent further incursions. Farmers relied on their own experiences and traditional knowledge to recognize signs and use traditional medicines to treat the pigs in the hope they would survive. Technicians and farmers corroborated the challenges of distance, poor communication, lack of knowledge, and coordination. Hence, passive disease surveillance was not happening in these locations. Such top-down approaches to animal disease management are common in developing contexts, particularly during outbreaks as donor funding is readily available (23). Active surveillance programs rarely consider livelihood factors or devote time and resources to educating farmers in husbandry and biosecurity techniques (23, 28). Passive surveillance requires community support, which in turn requires genuine engagement and closing of the disease information loop back to farmers.

The findings also showed that most technicians lacked experience and confidence with sampling or post-mortems so diagnostic training was an important component to underpin the pilot disease investigation and community engagement processes. The collection of samples from 27 pigs improved technician skills in taking skin, blood, and fecal samples; and doing post-mortems. The diagnostic results gave valuable information on ASF prevalence and associations with bacterial and parasite infections. Combining the passive surveillance disease investigation with the pilot community engagement program improved the knowledge, motivation, and confidence of government staff and farmers. Regular interactions during sampling and farmer training events created mutual understanding and joint learning between staff and communities. The process of engaging households in social learning (learning on the job with staff and other farmers) built relationships and trust. The credibility of veterinary technicians improved and gave them more confidence to work with communities. Barnes et al. (9) reported similar benefits from engaging farmers and technicians in feeding trials and biosecurity practices in Timor-Leste. Chenais et al. (22) and Aliro et al. (26) recommended greater support for community education and participatory methods that take into account local social and cultural contexts.

The disease investigation results highlighted the need for integrated husbandry approaches to disease management. Evaluation of the farmer training in all aspects of pig health and husbandry showed moderate increases in farmer knowledge. In a short timeframe, it also led to improving pig management practices by some farmers. Farmers felt supported because all aspects of pig husbandry were addressed and they reported being more willing to report dead or sick pigs. However, some biosecurity and feed measures (e.g., buying pen materials and dried feed stuffs) have structural and financial constraints for households that no amount of knowledge increase will change. Chilundo et al. (28) also found that a lack of basic resources for Mozambique pig farmers prevented them from total confinement of pigs. In Zambia, pig owners had many socioeconomic reasons for not confining pigs and accepted the health risks of porcine cysticercosis (25). Cultural habits, taboos, and poverty were factors limiting the implementation of ASF control measures in Uganda (29).

We conclude that the potential for improved passive disease surveillance and pig husbandry can be realized by engaging communities in productive practices in Timor-Leste. Recommendations for veterinary services in Timor-Leste are to conduct community engagement and diagnostic training in the remaining districts with government technicians and farmers. Animal science and animal health graduates from local universities should also be included so they can learn the methods and build experience working with farmers. Scaling out the approach would enable greater breadth and depth in evaluating impacts on pig health and livelihoods over a longer period of time.

The project relied on qualitative methods so was limited in sample sizes for statistical analysis. Future research could study potential correlations and relationships between pig herd sizes, farmer learning, and pig health outcomes. The benefits and interactions of active surveillance and passive surveillance methods in controlling pig diseases would be informative in terms of ongoing investments.

## Data availability statement

The raw data supporting the conclusions of this article will be made available by the authors, without undue reservation.

## Ethics statement

The studies involving human participants were reviewed and approved by University of Sydney Human Research Ethics Committee (Project number 2020/122). The participants provided their written informed consent to participate in this study. Written informed consent was obtained from the individual(s) for the publication of any potentially identifiable images or data included in this article.

## Author contributions

JM, PH, J-AT, OM, JJ, and HD conceived and designed the study. OM, HD, and AP conducted the fieldwork. OM, HD, and JM analyzed the social data. AF and PH analyzed the sample diagnostic data. FC, PH, and OM conducted the diagnostic training and sample preparation. OM and JM conducted the community engagement training. JM, PH, J-AT, ST, AP, and OM prepared the draft manuscript. All authors reviewed and edited the manuscript and have read and approved the final manuscript.
